# A Narrative Review of Home Enteral Nutrition in Australia with a Focus on Blended Tube Feeding

**DOI:** 10.3390/nu17060931

**Published:** 2025-03-07

**Authors:** Lina Breik, Lisa A. Barker, Judy Bauer, Zoe E. Davidson

**Affiliations:** Department of Nutrition, Dietetics and Food, Monash University, Notting Hill, VIC 3168, Australia; lisa.barker@monash.edu (L.A.B.); judy.bauer@monash.edu (J.B.); zoe.davidson@monash.edu (Z.E.D.)

**Keywords:** home tube feeding, home enteral nutrition, nutritional support, blended tube feeding, narrative review

## Abstract

Enteral nutrition, commonly known as tube feeding, is a life-sustaining intervention for individuals who cannot meet their nutritional needs orally due to medical conditions affecting swallowing, digestion, or nutrient absorption. Since its introduction in the 1970s, home enteral nutrition (HEN) has enabled the safe delivery of complete or supplemental nutrition in community settings, enhancing both quality of life and healthcare outcomes. The HEN landscape in Australia is rapidly evolving, driven in part by the renewed interest in blended tube feeding (BTF). This narrative review explores the current state of HEN care and BTF support in Australia, focusing on prevalence, funding models, provider and client perspectives, clinical guidelines, and advocacy efforts.

## 1. Introduction

**Figure 1:**
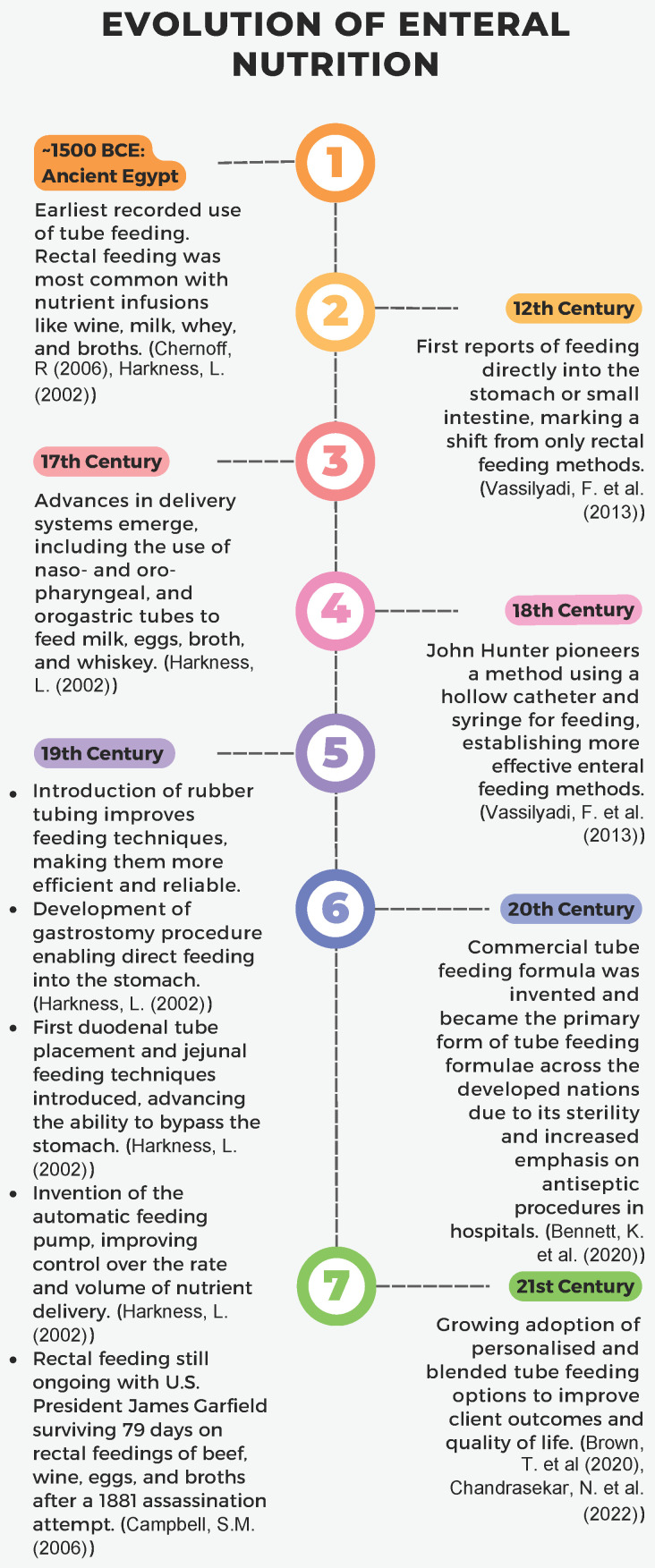
A timeline of the evolution of enteral nutrition [2,3,4,5,6,7,8].

Enteral nutrition, commonly known as ‘tube’ feeding, is a vital intervention for restoring and maintaining the health of those unable to meet their nutritional needs orally due to medical conditions affecting swallowing, digestion, or nutrient absorption [1]. The origins of enteral nutrition, as illustrated in Figure 1, date back over 3500 years to ancient Egyptian practices, with significant advances over the past century including delivery systems, the advent of commercial formulas and, more recently, homemade blended tube feeding [2,3,4,5,6,7,8].

Home enteral nutrition (HEN) is the method of delivering total or supplemental nourishment through a feeding tube in the residential or community setting, rather than in a healthcare facility [9,10]. HEN emerged as a reliable and efficient method for delivering essential nutrition in the 1970s, playing a crucial role in sustaining and prolonging lives [11].

HEN is often indicated for individuals with neurological conditions, head and neck cancer diagnoses, acquired brain injuries, or any other conditions that affect safe swallowing ability [11]. Furthermore, as chronic diseases continue to rise in Australia, their intersection with HEN is expected to grow, increasing the demand for enteral nutrition support [12,13]. Feeding tubes can be inserted for either short-term (4–6 weeks) or long-term purposes, depending on the individual’s medical and nutritional needs [14]. Nasogastric and orogastric tubes are used for short-term feeding needs and are commonly used in the hospital setting. In contrast, gastrostomies or jejunostomies are long-term feeding tubes that are more commonly found in the residential or community setting [15,16,17], and can be inserted either endoscopically, radiologically, or surgically [18,19].

Blended tube feeding (BTF), also referred to in the literature as ‘blenderized diet’ or ‘blended diet’, consists of everyday foods blended to a purée consistency and given through a feeding tube [20,21]. Individual food choices are shaped by a complex interplay of medical, cultural, religious, ethical, and personal factors, and tube feeding is no exception to these considerations, particularly in the home setting [7,13,22]. The landscape of HEN is evolving, with BTF becoming a significant part of the conversation. Before the 1970s, BTF was the primary approach to enteral nutrition before commercial formulas were available [23]. Today, BTF can be prepared at home or sourced commercially as blends made from real food.

As more individuals receive nutrition support at home [24], alternative approaches like BTF are reshaping clinical practice, patient preferences, and policy considerations. Understanding this evolving landscape is essential to ensuring equitable access, optimizing nutritional care, and supporting person-centred approaches.

## 2. Aims and Methodology

The primary objective of this narrative review is to provide a comprehensive overview of the evolving HEN and BTF landscape in Australia through an exploration of the prevalence of HEN and BTF, existing funding structures and provider and client perspectives. Additionally, this review will analyse emerging HEN and BTF guidelines, highlight key advocacy efforts to improve HEN services, and discuss future directions to support the integration and accessibility of HEN and BTF within the Australian healthcare system.

The methodology for this narrative review was informed by the SANRA checklist to ensure a structured approach (see Supplementary S1) [25]. A broad range of sources was consulted, including unpublished literature, funding documents, and professional association materials, to capture the most relevant insights. A snowballing approach was applied, beginning with a search for key terms such as ‘home enteral nutrition’ and focusing on Australian authors, followed by checking their reference lists for additional sources. Literature searches were conducted via Google Scholar, and further hand-searching of reference lists from known systematic literature reviews on BTF was performed to ensure comprehensive coverage. Additionally, insights from professional networks and associations contributed to the contextual understanding of HEN and BTF in Australia.

## 3. Prevalence of HEN and BTF in Australia

Limited data exist on the prevalence of home enteral nutrition (HEN) in Australia. In 2021, a comprehensive study was conducted to explore the characteristics of HEN services across Australia and New Zealand. This study utilized a cross-sectional survey distributed to lead clinicians in health services [26]. The majority of responses came from New South Wales (29%), Victoria (19%), and Queensland (13%). Fifty-seven percent of responses represented acute healthcare settings and the majority (95%) were from state government-funded hospitals.

From the 107 health services, the estimated number of HEN patients was approximately 7122 patients, with 65% (*n* = 4675) reliant on exclusive tube feeding [26]. The distribution of patients was nearly even between paediatric (*n* = 3725) and adult (*n* = 3397) categories, translating to about 234 people per million population receiving HEN in the community. This estimate is lower than the American prevalence of 1385 HEN patients per million, but higher than the British prevalence of 92 HEN patients per million [27,28].

Among paediatric HEN services, oncology (54%) was the most common specialty, followed by neurology (46%). For adult and combined services, head and neck conditions were predominant (61% and 67%, respectively). The most frequently used feeding tubes were gastrostomy tubes (74%), followed by nasogastric tubes (17%).

In terms of BTF, 40% of surveyed health services reported that their clients used BTF for some or all of their enteral nutrition needs. The prevalence was higher in predominantly paediatric services, with 76.9% (10 out of 13) reporting BTF use. However, BTF was also reported in adult services (21.7%, 10 out of 46), and combined adult and paediatric services (45.7%, 21 out of 46). In comparison, a prospective cross-sectional study conducted in the United States found that 55.5% of adults receiving HEN used BTF, with a median frequency of 4 days per week (range: 1–7 days). These Australian figures provide the only available estimates to date on BTF usage within the country.

Despite its scale, this single study looking at HEN prevalence in Australia has limitations. Its cross-sectional design and convenience sampling approach may introduce selection bias, as the majority of respondents were from the east coast of Australia and from acute state government-funded hospitals. This means minimal representation from private hospitals, clinics, community services, and disability services introducing selection. Nonetheless, it remains the largest and only study of HEN services to estimate prevalence across Australia and New Zealand to date.

Two other Australian studies have attempted to estimate HEN in New South Wales. Notably, they included patients on home oral nutrition support (81% and 76% of patients, respectively) within their HEN populations, whereas Flood et al. focused exclusively on patients requiring enteral ‘tube’ feeding [26,29,30]. This inconsistency in defining the HEN population complicates comparisons across studies but also highlights a barrier to accurately estimating HEN prevalence in Australia. Without a standardized definition, the true scope of HEN use remains unclear, leaving policymakers and healthcare providers without the data needed to effectively allocate resources and plan services.

It is also unclear whether HEN prevalence is increasing in Australia. Victorian data suggest a substantial rise in HEN usage over the past two decades, from 400 individuals estimated in 1999 to 1745 in 2021 [26,31]. The lack of a national HEN registry in Australia limits the ability to accurately assess prevalence and incidence of existing and new patients requiring HEN, respectively. Clinical quality HEN registries in countries, such as the UK and Spain, have proven effective in collecting standardized data to monitor quality of care, assess access, and drive improvements, such as equitable funding, to HEN delivery through benchmarking [32,33,34]. Establishing a similar registry in Australia could enhance our understanding and management of the HEN population. As questioned seven years ago in a commentary on Faruquie et al.’s evaluation of HEN services in New South Wales: Is it time for Australia to follow suit? [35].

## 4. Funding of HEN and BTF in Australia

Financial and clinical support for individuals who require HEN in Australia come from the state government and/or the federal government.

State government-funded initiatives supporting HEN include programs offered through public hospitals, motor vehicle accident insurance schemes (e.g., the Transport Accident Commission [TAC] in Victoria), and Home Care Packages (HCP) for older adults [36]. However, financial support varies significantly across Australian states [26,29,30,31,37]. For example, in New South Wales, clients contribute to the monthly cost of tube-feeding supplies provided by state-funded hospitals. In contrast, hospitals in Victoria, Western Australia, and Queensland cover these costs in full [26,38,39,40]. Even within the same state, funding support from different hospitals is inconsistent. Research by Faruquie 2016 [30] and Tang 2019 [29] across several hospitals in New South Wales revealed that these disparities often attributed to resource limitations, which is also a barrier to consistent service provision.

Federal government-funded initiatives, such as the National Disability Insurance Scheme (NDIS), introduced in 2013 and hailed as Australia’s most significant social policy reform since Medicare in 1984 [41], aim to provide consistent support across states. However, a systematic review by Carey and colleagues (2021) highlighted challenges faced by users navigating the NDIS. Participants frequently described the application process as administratively burdensome, time-consuming, and complicated by inconsistent and inaccurate information [42]. The review also revealed that the scheme remains highly inequitable in terms of funding provision and spending practices. A key issue is the stringent gatekeeping around what qualifies as ‘reasonable and necessary support’, which ultimately determines whether participants receive adequate funding.

Some individuals who are home tube-fed do not qualify for state or federal funding support, resulting in out-of-pocket costs which can be substantial [43]. The actual expense of HEN in Australia remains unclear due to conflicting estimates. Earlier studies from 2021 report tube-feeding consumables costing between AUD 0 and AUD 341 (USD 0–USD 225) per month per client [26]. In contrast, a 2023 observational study of an NDIS HEN service operating out of a major acute paediatric health service described median costs of AUD 1124 (USD 742) per month, with a range from AUD 197 to AUD 3681 (USD 130–USD 2430) per month [37]. These variations highlight important gaps in understanding the cost of HEN. Comprehensive and contemporary research describing the financial burden and service provision challenges faced by home tube-fed individuals in Australia is needed.

Individuals who use BTF face additional funding challenges within the current HEN funding landscape. BTF funding policies across Australia are inconsistent or do not exist. For example, the Victorian Government HEN Program and the Victorian Transport Accident Commission explicitly exclude BTF limiting funding to a specific list of approved formulas [40,44]. Thus, clients choosing or needing to use BTF (for allergy or intolerance reasons) must cover the costs of all required tube-feeding consumables. Flood et al. found that up to 28% of government health services report limiting or ceasing clinical and financial support for clients who choose BTF [26]. Similarly, the Australian Government Home Care Package (HCP) and the Department of Veterans’ Affairs (DVA) provide no specific support for BTF [45,46]. While the NDIS offers some flexibility by assessing requests on a case-by-case basis [47], the process is complex. This fragmented approach to BTF funding contradicts the Australian Commission on Safety and Quality in Healthcare’s Principles of Care, which emphasize client involvement in decision-making and the right to support accordingly—formula choice for tube feeding is no exception [48].

## 5. Provider Perspectives of HEN and BTF

Research on the perspectives of HEN providers in Australia is limited. Identified challenges from existing research include inadequate funding for staff, lack of a multidisciplinary team approach, navigating inconsistent care across different settings, and a shortage of staff with specialised HEN expertise [26,29,30]. Provider satisfaction was found to be highest regarding training, yet notably lower for staffing resources, reflecting these workforce challenges [26]. Hospitals with a dedicated HEN dietitian or coordinator are significantly more likely to have structured policies in place, ensuring consistency in service provision, patient selection, monitoring, and termination of HEN [29,30]. However, existing studies have primarily focused on providers working within traditional hospital-based HEN models, overlooking those operating in NDIS-funded disability-specific community services. Research on HEN provider experiences within the NDIS is scarce. One of the few studies in this space, Comito et al. (2023), explored the HEN provider experience of transitioning clients from hospital-based HEN care to an NDIS outpatient service. The study highlighted the administrative burden on HEN providers, noting that clinicians spent substantial time preparing detailed, individualized support letters to justify fair and reasonable annual HEN funding. This highlights the bureaucratic challenges of working within the NDIS system [37].

Provider perspectives around BTF have been explored primarily in developed countries. These include perceived limitations in competence, insufficient awareness of professional guidelines, and inadequate training [49,50,51]. In Australia, Reilly et al. (2024) conducted a cross-sectional survey of 89 health professionals and identified key barriers to supporting BTF as clinician time constraints, resource limitations, and the absence of formal guidelines [52]. Some providers also expressed concerns about potential risks, such as poor growth or weight loss in patients. The study found associations between BTF training and clinical practice, underscoring the importance of education, institutional policy, and resource availability in promoting evidence-based BTF practices. However, a limitation of the study is that it does not explicitly report its response rate, making it difficult to assess if the sample represents HEN dietitians. With only 89 respondents, selection bias may be present, as those with prior interest in BTF may have been more likely to participate so responses may not fully capture the diverse perspectives of health professionals across different settings. Reporting the total number of surveys distributed and addressing potential non-response bias would strengthen the study’s validity. Future research should aim to improve participation through broader recruitment strategies and targeted outreach to include a range of health professionals and settings.

## 6. Client Perspectives of HEN and BTF

From the client perspective, a 2022 scoping review of 19 studies across seven countries (Singapore, United Kingdom, United States, Sweden, Northern Ireland, Germany, and France) identified several barriers to HEN care. These included insufficient education at hospital discharge, lack of support from knowledgeable clinicians, and limited access to community-based care, all of which negatively impacted quality of life [53]. The authors also found some key enablers of HEN care from the client perspective, such as having a dedicated HEN team within their health network, the provision of education on practical handling and daily care of the feeding tube management of complications, and greater specificity and concreteness in educational material regarding where to seek support from clinicians [53]. Beyond logistical challenges, research also highlights the social and emotional toll of HEN. A qualitative study from the United Kingdom found that adults with head and neck cancer on HEN experienced guilt and isolation, which strained family relationships [54]. Similarly, a Canadian study reported that caregivers of individuals dependent on tube feeding faced significant physical, emotional, and social burdens [55]. Despite these challenges, several enablers were identified in these two studies: (1) an understanding that the feeding tube offered an advantage when swallowing was not possible, ensuring adequate nutrition; (2) consistent support from family, friends, or the public; (3) maintaining a positive and resilient attitude; and (4) information and training tailored to the specific needs of both the client and their caregiver [54,55]. To our knowledge, there is no research on the client perspectives, barriers and enablers of HEN care in Australia, highlighting a gap in understanding their experiences and needs.

Regarding client perspectives on BTF, Trollip et al. (2019) investigated BTF use from a parental viewpoint in an Australian paediatric population (*n* = 12) [56]. Parents reported improvements in their children’s growth, gastrointestinal symptoms, and social inclusion as key benefits of BTF. The study also noted that many families accessed information online, emphasizing the need for health professionals to acknowledge this trend and provide open support, guidance, and monitoring to these families. However, parents identified several barriers to using BTFs, including the time required for careful planning and preparation, as well as challenges with travel due to storage and reheating needs. Concerns about potential tube blockages and the need for specialized equipment, such as gastrostomy extensions and appropriate blenders, were also highlighted. While this study provides valuable insights, the small sample size (*n* = 12) raises questions about the generalizability of its findings. A sample of this size is unlikely to capture the full spectrum of parental experiences and limits the applicability of conclusions to broader populations. Currently, no other Australian research has been published on provider and client perspectives on BTF, indicating a significant gap in the literature.

To enhance HEN care and BTF support, it is essential to capture the perspectives of both providers and clients in Australia. Such research could inform the development of locally targeted guidelines, practical resources, and evidence-based policies to address current gaps and improve care outcomes [57].

## 7. Guidelines on HEN and BTF in Australia

The development of guidelines is a complex process, shaped by a combination of evidence and practice considerations. A 2024 systematic review set out to systematically identify and summarize existing guidelines pertaining to the provision of HEN in adults and assess the quality of guidelines and their recommendations using internationally recognized quality assessment tools. They found 15 existing national and international HEN guidelines (two from Australia) [58]. Using the AGREE-REX tool, most guidelines were found to be of poor methodological quality, with low scores in ‘rigour of development’ and ‘applicability’. ‘Clinical Applicability’ was the highest scoring domain, while ‘Values and Preferences’ was the lowest scoring. The two guidelines from Australia included in the systematic review were both published by a New South Wales-based organization, The Agency of Clinical Innovation (ACI) [59,60]. Neither of their two documents address BTF. Additionally, The Australasian Society of Parenteral and Enteral Nutrition (AuSPEN) and Dietitians Australia have also published HEN-related guidelines, which did not meet the inclusion criteria for this review. This illustrates how the development of HEN guidelines has been completed by individual organizations rather than a unified, consistent approach [36,61,62].

As interest in BTF grows, professional organizations have developed guidelines to address its use and implementation. In 2021 AuSPEN published an Australian consensus statement to provide guidance on the use of BTF, reflecting the growing interest in this feeding approach. [61] Internationally, guidelines have also been developed by organizations such as the American Society of Parenteral and Enteral Nutrition (ASPEN) (2017, 2023), the European Society of Parenteral and Enteral Nutrition (ESPEN) (2022), and the British Dietetic Association (BDA, 2019) [11,21,63,64]. However, these organizations present differing narratives: ESPEN and AuSPEN take a cautious stance emphasizing risk mitigation, whereas ASPEN and BDA focus on implementation, demonstrating a more accepting approach. The divergence in these positions reflects the inconsistent evidence base informing their recommendations. For example, within the existing BTF evidence for adults, some systematic reviews suggest potential benefits, such as improved patient acceptability and relief of gastrointestinal symptoms [65,66,67]. However, concerns about microbial contamination and nutritional adequacy remain notable [68,69]. Additionally, the overall evidence base is of low certainty due to methodological limitations that complicate the interpretation of study findings. As highlighted in the most recent systematic review, these limitations include the lack of a standardised BTF definition across studies, inconsistencies in recipe formulation and nutritional equivalence, and the absence of validated tools to assess outcomes such as nutritional status or quality of life [67]. These challenges make it difficult to draw definitive conclusions about the true impact of BTF.

## 8. Advocacy of HEN and BTF in Australia

Advocacy for HEN service provision at a national level has been at the forefront in Australia for nearly two decades. Dietitians Australia, the leading professional body representing over 9000 nutrition professionals, has been at the forefront of these efforts. In 2009 and 2015, the organization presented initiatives to the Australian Health Ministers Advisory Council, proposing the establishment of a national HEN program to address service delivery gaps and improve support for clients [70,71].

More recently, HEN was highlighted as a key priority in Dietitians Australia’s 2023 pre-New South Wales election submission. This advocacy focused on improving funding for HEN clients relying on supplies from state-funded hospitals in New South Wales, aiming to reduce inequities in access and affordability [72].

For BTF, the 2021 AuSPEN consensus statement and its accompanying resource mark Australia’s first formal guidance on this emerging area of practice [61]. Additionally, Reilly et al.’s study published in 2024 on Australian clinicians’ perceptions of BTF has contributed valuable insights, helping to build a foundation of knowledge and awareness [52]. Together, these initiatives represent meaningful steps toward advancing both HEN and BTF services, though continued advocacy and research remain essential.

**Figure 2:**
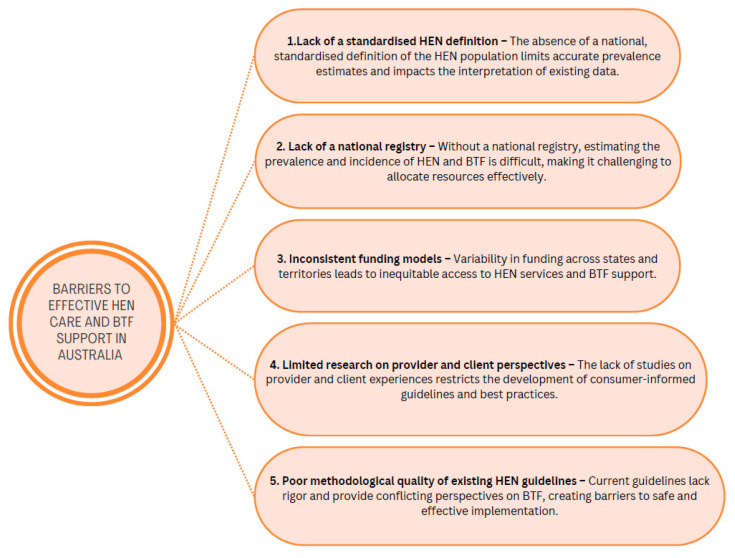
Barriers to effective home enteral nutrition (HEN) care and blended tube-feeding (BTF) support in Australia.

However, despite attempts of advocacy for a national HEN program over the past two decades, improvements in HEN care delivery remain hindered by persistent barriers, as summarised in Figure 2.

## 9. Implications for HEN and BTF Practice and Research

The evolution of HEN and BTF in Australia reflects a global shift toward more personalized and person-centred nutrition support. To enhance HEN care, clinicians must develop a thorough understanding of relevant funding bodies to support clients to access enteral nutrition resources. Awareness of the growing evidence base for BTF is essential to ensure informed decision-making and individualized care planning. As the landscape of HEN continues to evolve, clinicians should remain adaptable, engaging in continuous education and collaboration to align with emerging best practices.

To address the barriers outlined Figure 2, future research should investigate the real-world application of BTF in various care environments, including community-based settings, could provide valuable insights into current practices, challenges, and outcomes. Additionally, research exploring the long-term financial, clinical, and psychosocial impacts of HEN can inform policy development and service improvements. Strengthening the evidence base in these areas will support the continued evolution of HEN and BTF, ultimately improving patient care and accessibility.

This review is limited by several factors, including its predominantly Australian perspective, reliance on a narrative approach, and the age of published studies cited. Additionally, existing research on HEN is constrained by small sample sizes in predominantly hospital populations, limiting the generalizability of challenges and barriers to broader community and private-sector settings. These limitations highlight the need for further research to provide a more comprehensive understanding of HEN care and BTF across diverse healthcare environments.

## 10. Conclusions

This narrative review has examined the evolving landscape of HEN and BTF in Australia. Evidence suggests that over 7000 adults rely on HEN, yet access to funding and support remains inconsistent nationwide. Additionally, there is limited understanding of client and provider experiences, and high-quality guidelines are lacking. Substantial gaps persist in policy, funding, and service delivery.

Addressing these challenges requires a collective effort from stakeholders, including clients, caregivers, healthcare providers, and policymakers, to advocate for cohesive national policies, equitable funding models, and evidence-based guidelines that integrate both clinical expertise and lived experiences. Only through sustained collaboration and advocacy can we bridge the gaps in HEN care, ensuring that all Australians receive safe, effective, and dignified nutritional support that enhances their quality of life and upholds their right to informed healthcare decisions.

## Data Availability

No new data were created or analyzed in this study.

## Supplementary Materials

The following supporting information can be downloaded at: https://www.mdpi.com/article/10.3390/nu17060931/s1, Supplementary S1—SANRA Checklist for Narrative Reviews.
